# Interneuronal modulations as a functional switch for cortical computations: mechanisms and implication for disease

**DOI:** 10.3389/fncel.2024.1479579

**Published:** 2025-01-23

**Authors:** Yann Zerlaut, Alexandra Tzilivaki

**Affiliations:** ^1^Sorbonne Université, Institut du Cerveau - Paris Brain Institute - ICM, Inserm, CNRS, APHP, Hôpital de la Pitié Salpêtrière, Paris, France; ^2^Charité-Universitätsmedizin Berlin, corporate member of Freie Universität Berlin, Humboldt-Universität Berlin, and Berlin Institute of Health, Neuroscience Research Center, Berlin, Germany; ^3^Einstein Center for Neurosciences, Chariteplatz, Berlin, Germany; ^4^NeuroCure Cluster of Excellence, Chariteplatz, Berlin, Germany

**Keywords:** interneuron, vasoactive intestinal peptide, NDNF interneurons, cortical computation, cortex, inhibition

## Abstract

Understanding cortical inhibition and its diverse roles remains a key challenge in neurophysiological research. Traditionally, inhibition has been recognized for controlling the stability and rhythmicity of network dynamics, or refining the spatiotemporal properties of cortical representations. In this perspective, we propose that specific types of interneurons may play a complementary role, by modulating the computational properties of neural networks. We review experimental and theoretical evidence, mainly from rodent sensory cortices, that supports this view. Additionally, we explore how dysfunctions in these interneurons may disrupt the network’s ability to switch between computational modes, impacting the flexibility of cortical processing and potentially contributing to various neurodevelopmental and psychiatric disorders.

## Introduction

The mammalian brain’s ability to adapt to varying cognitive states and environmental demands is crucial for survival. This flexible neuronal processing allows for seamless adaptation, a task that remains challenging for artificial neural networks, despite their ability to match human performance in many areas. Understanding how humans and mammals, such as rodents, achieve this adaptability with ease is still a significant scientific challenge. In particular, it remains unclear which neurons within the cortical network are primarily responsible for such rapid adaptations and the cellular mechanisms that enable this flexibility. To fully grasp how successful modulation of cortical computations operates, a detailed understanding from the cellular to the systems level, supported by theoretical validation, is essential.

Since the discovery of GABAergic synaptic transmission in the brain (Crawford and Curtis, 1964; Krnjević and Phillis, 1963; Krnjević and Schwartz, 1967), inhibition was initially perceived as a simple counterbalance to excitatory glutamatergic transmission. However, subsequent research revealed far more complex roles for inhibition (Adesnik et al., 2012; Bishop et al., 1973; Chrobak and Buzsáki, 1996; Constantinidis et al., 2002; Monier et al., 2003; Pouille and Scanziani, 2001; Pouille et al., 2009; Wehr and Zador, 2003), leading to intense investigation into the multifaceted functions of inhibitory interactions (Roux and Buzsáki, 2015; Tremblay et al., 2016).

The rationale behind inhibitory interactions in the brain is not immediately apparent. Synaptic inhibition requires significant metabolic costs (Buzsáki et al., 2007), whereas the brain has more cost-effective mechanisms (e.g., refractoriness, saturation, adaptation, depression) that can prevent runaway excitation due to recurrent feedback loops. Moreover, inhibition is not strictly necessary for designing efficient signal processing systems; artificial neural networks, which lack dedicated inhibitory populations, still achieve human-level performance in many tasks (Mnih et al., 2015). One might then wonder why inhibition exists in biological systems. A key difference between artificial and biological networks is that the former lacks the generalization and flexibility (Sinz et al., 2019) observed in the latter. This suggests that such features in the mammalian brain might be mediated by inhibitory interactions. In this perspective, we propose that the flexible modulation of cortical processing is closely tied to inhibitory control of cortical dynamics.

Inhibition in the mammalian cortex is characterized by remarkable diversity in morphological, electrophysiological, molecular, and connectivity properties across brain areas (Ascoli et al., 2008; Defelipe et al., 2013; Klausberger and Somogyi, 2008; Pelkey et al., 2017; Tzilivaki et al., 2023). This diversity likely supports a division of labor in cortical processing, with different inhibitory populations contributing to distinct network functions (Kepecs and Fishell, 2014; Tremblay et al., 2016; Wang et al., 2004). Here, we hypothesize that a specific subset of inhibitory interneurons plays a crucial role: modulating the computational properties of cortical networks by controlling recurrent dynamics. We focus on rodent sensory systems as a model for cortical computations and explore how dysregulation of this mechanism might explain certain cognitive disorders.

## Classical roles of inhibition in cortical processing

To emphasize the specific viewpoint of this perspective, we first outline the established functional roles of inhibitory interneurons. These roles can be broadly categorized into two main functions: (1) the regulation of network stability and rhythmicity, and (2) the refinement of cortical representations.

The primary function historically attributed to synaptic inhibition is to stabilize network dynamics, preventing runaway activity that could result from the positive feedback loops of glutamatergic excitatory recurrence. Early evidence supported the notion that a network devoid of inhibition would exhibit pathological activity patterns. Convulsant drugs, such as bicuculline, picrotoxin, and penicillin, which induce acute epileptic seizures, were also found to strongly suppress inhibitory GABAergic synaptic transmission (Dingledine and Gjerstad, 1980; Hablitz, 1984; Wong and Prince, 1979). Theoretical work has also demonstrated that synaptically coupled networks benefit greatly from an architecture that mixes excitatory and inhibitory units. In configurations of symmetric interactions, excitatory/inhibitory networks tend to stabilize in a regime where excitatory and inhibitory synaptic currents are balanced (Renart et al., 2010; van Vreeswijk and Sompolinsky, 1996). This dynamic balance linearizes responses to incoming inputs, and gives remarkably fast processing capabilities to the network (van Vreeswijk and Sompolinsky, 1996). This balance between excitation and inhibition has since been observed experimentally (Dehghani et al., 2016; Haider et al., 2006) and is now considered a hallmark of cortical dynamics (Okun and Lampl, 2009).

Another key computational benefit of fast synaptic inhibition is its ability to generate rhythmic activity within networks (Chrobak and Buzsáki, 1996; Sohal et al., 2009). Inhibitory interactions create alternating phases dominated by either inhibition or excitation, leading to collective oscillations at the network level (Allen and Monyer, 2015; Buzsáki and Wang, 2012; Topolnik and Tamboli, 2022; Tzilivaki et al., 2023). These brain oscillations, and their modulation, are thought to support various cognitive processes (Buzsáki and Draguhn, 2004) such as representation through phase information (O’Keefe and Recce, 1993), perceptual grouping (Singer, 1999), memory consolidation (Cummings and Clem, 2019; Cummings et al., 2021; Lucas and Clem, 2018; Tzilivaki et al., 2023), and inter-areal communication (Fries, 2015). In this way, inhibition plays a critical functional role in cognition by regulating neural rhythmicity (Tzilivaki et al., 2023).

In recent decades, another crucial role of inhibitory interactions in cortical processing has been uncovered: the fine-tuning of cortical representations of the external world to enhance their fidelity and optimize the cortex’s encoding capabilities. For instance, in the visual system of mammals, cortical inhibition has been shown to support visual processing by (i) sharpening sensory representations temporally (David et al., 2004; Priebe and Ferster, 2005; Ringach et al., 1997), (ii) reducing spatial redundancy through lateral inhibition (Chemla et al., 2019; Miller, 2003), and (iii) increasing sparseness through recurrent inhibition (Haider et al., 2010; King et al., 2013; Vinje and Gallant, 2000). By ensuring temporal precision and reducing redundancy in sensory representations, inhibition plays a critical role in shaping cortical processing.

## Properties of specific interneuronal subpopulations suggest non-classical roles

Recent findings suggest that the classical roles attributed to inhibitory interneurons may not apply uniformly across all inhibitory populations, pointing to potentially different functions for some subpopulations in neocortical processing. This hypothesis is supported by both anatomical and functional characterizations.

**Figure 1:**
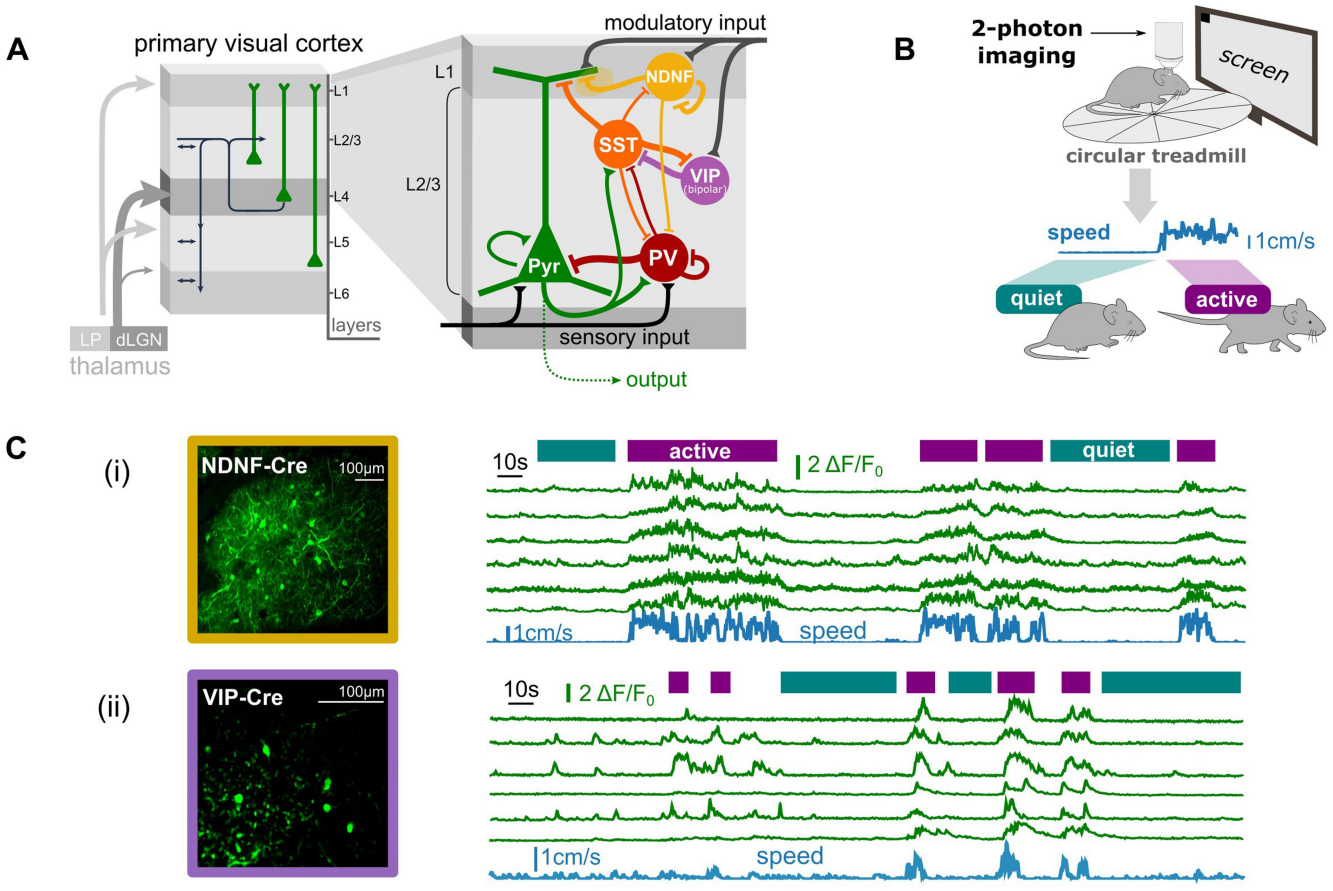
Anatomical and functional properties of specific interneuronal subpopulations suggest non-classical inhibitory roles. **(A)** Schematic of the cortical networks in the visual cortex with a zoom on the supragranular networks (layer 1 and 2/3) network with some of its molecularly-defined inhibitory subpopulations (PV: Parvalbumin positive INs, SST: Somatostatin positive INs, VIP: Vasointestinal positive peptide INs, NDNF: Neuron-derived neurotrophic factor positive INs). **(B)** Schematic of the experimental setup to record interneuronal activity during spontaneous behavior (here running/walking vs. quiet) using two-photon imaging in head-fixed mice. **(C)** Neuronal activity of the VIP (ii) and NDNF (iii) interneuronal populations in awake behaving mice with simultaneous monitoring of behavioral states (“active” vs. “quiet”) extracted from the locomotion speed (blue trace). Note the strong correlation between running and interneuronal activity in those populations. Data from (i) are adapted from van Velze et al. (2024) and data in (ii) are unpublished data (Zerlaut, Van Velze, Bacci, Rebola).

Figure 1A presents a simplified diagram of the supragranular network of the cortex (i.e., layer 1 and layer 2/3), depicting interneurons within the largest, mostly non-overlapping, molecularly-defined inhibitory subpopulations, along with the excitatory pyramidal cell population (see Tremblay et al., 2016 for a perspective). In layer 1 (L1), neuron-derived neurotrophic factor positive (NDNF+) interneurons constitute approximately 70% of the L1 population (Abs et al., 2018; Schuman et al., 2019). In layer 2/3 (L2/3), PV+ interneurons make up about 35% of the interneurons, somatostatin-positive (SST+) interneurons around 20%, and vasoactive intestinal peptide-positive (VIP+) interneurons another 20% (Pfeffer et al., 2013).

The PV+ population in L2/3, largely mainly composed of basket cells, strongly targets the perisomatic compartment of pyramidal cells (Kawaguchi, 1997; Figure 1A), granting it significant inhibitory control over the local excitatory population. PV+ basket cells are prototypical of fast, potent inhibition, with well-established roles in controlling network stability, rhythmicity, and the temporal regulation of neuronal representations (Lourenço et al., 2020; Tremblay et al., 2016). The SST+ population in L2/3, primarily composed of Martinotti cells, also innervates pyramidal cells but targets their apical dendrites (Kawaguchi and Kubota, 1996; Figure 1A). Recent research has shown that the SST+ population plays a crucial role in shaping cortical representation by implementing lateral suppression (Adesnik et al., 2012) and regulating specific rhythms in the mouse sensory cortex (Chen et al., 2017; Veit et al., 2017). Thus, both PV+ and SST+ interneurons exhibit properties that align with the classical roles of inhibition.

In contrast, interneurons from the NDNF+ and VIP+ populations display properties that challenge the classical view of inhibition. First, the largest fraction of VIP+ interneurons (“bipolar VIP+” cells: ~60% of VIP+ cells in layer 2/3), for instance, does not directly contact excitatory cells (Pfeffer et al., 2013; Figure 1A); instead, it primarily targets inhibitory SST+ cells, forming a disinhibitory circuit that only indirectly influences the local excitatory network. Next, the NDNF+ population targets the apical tufts of pyramidal cells (Schuman et al., 2019; Figure 1A) predominantly via slow GABAb-mediated currents (Oláh et al., 2009; Schuman et al., 2019; Tamás et al., 2003) as well as via GABAa currents exhibiting slow responses due to their subunit composition (Szabadics et al., 2007). Additionally, the NDNF+ population inhibits itself (Fang et al., 2020; Schuman et al., 2019; Figure 1A) as well as the PV+ population, forming another disinhibitory circuit (Cohen-Kashi Malina et al., 2021; Letzkus et al., 2011; Takesian et al., 2018; Figure 1A). These populations lack the fast, direct inhibition seemingly required to control the stability and rhythmicity of cortical dynamics. Functionally, their neuronal activity is more strongly correlated with the animal’s behavioral state than with sensory-evoked signals, as demonstrated for both the VIP+ (Fu et al., 2014; Millman et al., 2020) and NDNF+ populations (Cohen-Kashi Malina et al., 2021). For example, in awake mice running on a circular treadmill (Figure 1B), the neural activity of both populations shows a strong correlation with the locomotion signal (Figure 1C), which serves as a proxy for the animal’s global arousal state. These populations are heavily innervated by external neuromodulatory inputs that convey arousal modulation signals to the local cortical network (Hattori et al., 2017; Figure 1A).

In summary, these lines of evidence indicate that both bipolar VIP+ and NDNF+ interneurons (1) do not exert a clear, fast inhibitory effect on excitatory cells, limiting their ability to control the stability and rhythmicity of local network activity, and (2) are primarily activated by exogenous, non-sensory signals rather than sensory-evoked activity in the sensory cortex, suggesting they do not play a major role in refining cortical representations.

## Inhibitory modulations can change the computational properties of cortical networks

The strong involvement of VIP+ and NDNF+ interneurons in modulating network dynamics based on the animal’s state (Figure 1C) suggests they may play a crucial role in this aspect of cortical processing. Recent studies have demonstrated significant modulation of excitatory and inhibitory dynamics in the sensory cortex of rodents, closely linked to behavioral and arousal states (McGinley et al., 2015a; Musall et al., 2019; Pakan et al., 2016; Stringer et al., 2019). Notably, transitions between behavioral states and their corresponding modulations of local cortical dynamics have a profound impact on signal processing within the sensory cortex (Busse et al., 2017; McGinley et al., 2015a). For instance, in the mouse visual cortex, the aroused state associated with locomotion significantly increases visual responses (Niell and Stryker, 2010), broadens orientation tuning (Reimer et al., 2014), and alters performance in detection tasks (Neske et al., 2019). This raises intriguing questions: Could the recruitment of specific interneuronal populations underpin these changes in cortical signal processing? And if so, what are the underlying mechanisms?

A well-established type of modulation in cortical networks involves quantitative changes. In the visual cortex, for example, the modulation of sensory-evoked response gain associated with locomotion (Ferguson and Cardin, 2020; Niell and Stryker, 2010) has been linked to VIP-mediated disinhibition (Fu et al., 2014). During periods of high arousal, such as when an animal is running, nicotinic input from the basal forebrain activates VIP+ interneurons. These, in turn, inhibit SST+ interneurons (Figure 1A), effectively removing the “blanket of inhibition” over pyramidal cells (PCs) and allowing for enhanced responses in these excitatory neurons (Fu et al., 2014; Karnani et al., 2016). Similarly, the NDNF+ population modulates gain in the visual cortex through a parallel pathway (Fang et al., 2020). Neuromodulatory input activates NDNF+ interneurons during high arousal, which then inhibit PV+ interneurons (Figure 1A), thereby reducing somatic inhibition on pyramidal cells and enhancing visually-evoked responses (Cohen-Kashi Malina et al., 2021).

**Figure 2:**
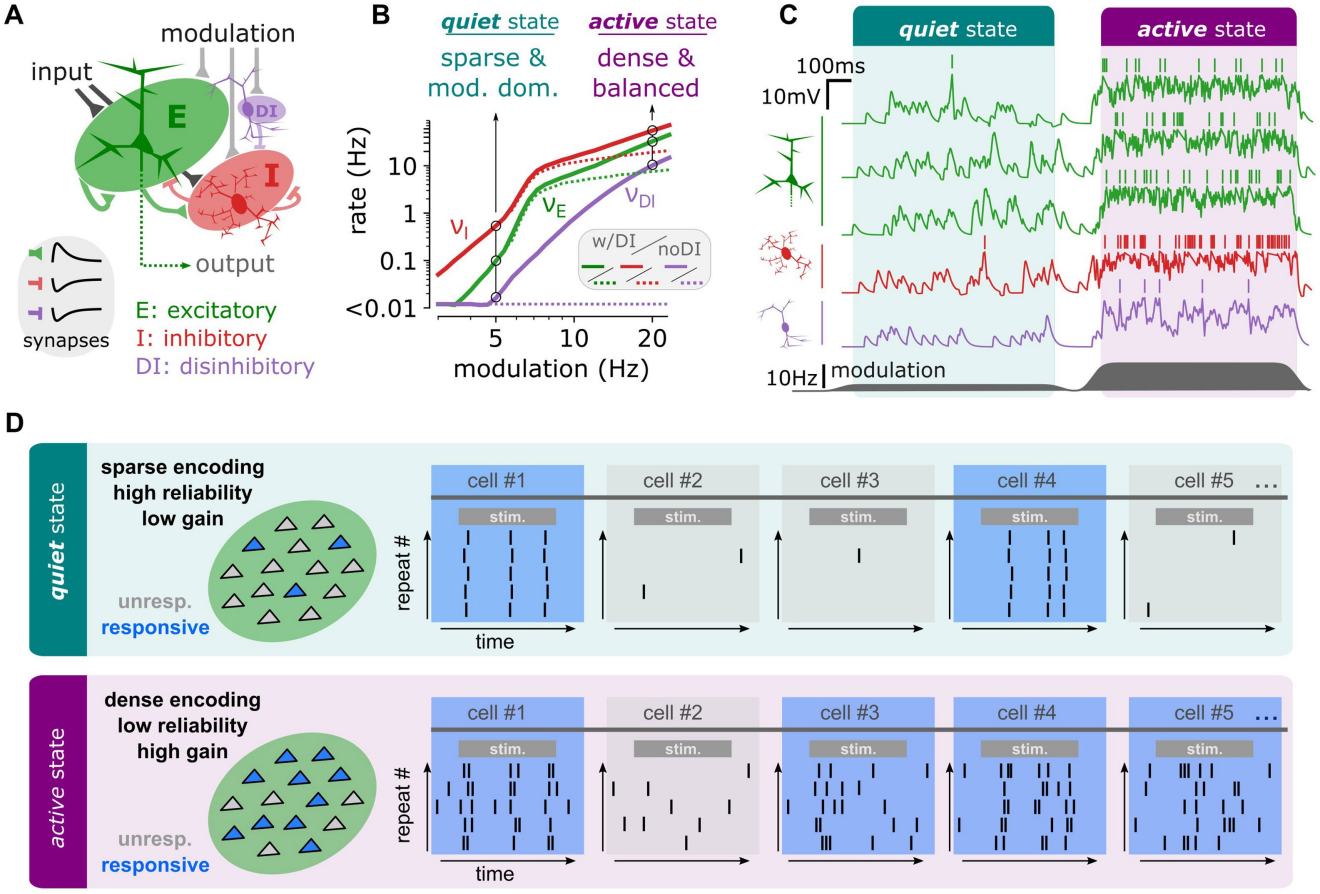
In a simplified model of the cortical circuit, disinhibitory recruitment can qualitatively change the network’s computational properties. **(A)** Schematic of the network model. The model is made of three populations of sparsely connected spiking units: Excitatory (E, green), Inhibitory (I, red) and Disinhibitory (DI, purple) integrate-and-fire neuronal models randomly interconnected by excitatory (E) or inhibitory (I, DI) synapses. **(B)** Stationary firing rate activity of the different network populations (E: green, I:red, DI: purple) as a function of the modulatory activity. At low levels of modulation (5 Hz), we highlight a regime of sparse and modulation-dominated activity corresponding to the dynamics observed in the *quiet* state. At high levels of modulation (20 Hz), we highlight a regime of dense and recurrently balanced activity corresponding to the dynamics observed in the active state. **(C)** Network activity in terms of membrane potential dynamics in a set of example neurons from each population (E: green, I: red, DI: purple). The transition between is achieved dynamically by changing the level of modulation (dark grey, bottom). **(D)** Schematic of the specific computational properties associated to each regime in network activity. The *quiet* state corresponds to a computational mode of sparse encoding, low gain, and high reliability while the *active* state corresponds to a computational mode of dense encoding, high gain and low reliability. Panels **(A–C)** are reprinted from Zerlaut et al. (2019).

Beyond quantitative changes, recent theoretical research has revealed that inhibitory circuits can profoundly alter the computational properties of cortical networks (Zerlaut et al., 2019). In a simplified network model (Figure 2A) that mimics the distinct network states observed at different arousal levels in the mouse sensory cortex (McGinley et al., 2015b; Zerlaut et al., 2019, 2022), it was shown that varying levels of an afferent modulatory variable—representing neuromodulatory effects associated with arousal changes—could induce different network states. At low modulation levels, the network exhibited an afferent-dominated regime (analogous to the “quiet state” *in vivo*), characterized by sparse activity. At high modulation levels, it shifted to a recurrent-dominated regime (similar to the “active state” *in vivo*), marked by dense activity and balanced synaptic currents (Figure 2C). Notably, the transitions between these distinct activity regimes were greatly facilitated by the introduction of a disinhibitory circuit, resembling the VIP+ and NDNF+ populations discussed earlier (Figure 2B).

Crucially, these different network activity regimes led to markedly different processing of incoming stimuli. In the quiet state, stimulus encoding was sparse (involving a small number of neurons), highly reliable across trials, and characterized by relatively low gain (Figure 2D; Zerlaut et al., 2019). In contrast, in the active state, stimulus encoding was dense (involving many neurons), less reliable due to variability in the recruited neurons across trials and exhibited a remarkably high gain (Figure 2D). The quiet state was found to be optimal for encoding complex synaptic patterns, while the high sensitivity of the active state favored the detection of weak stimuli (Zerlaut et al., 2019). Since these distinct network states—and their associated computational properties—relied on the presence of a disinhibitory circuit, this theoretical analysis suggests a novel role for specific inhibitory populations, such as VIP+ and NDNF+ interneurons: facilitating shifts in the computational mode of the cortical network.

## Implications for interneuronal-related diseases

The hypothesis that specific cortical interneuron types, such as bipolar VIP+ and NDNF+ interneurons, might play a potential role in modulating cortical network dynamics suggests that dysfunction in these neurons could potentially impair the network’s ability to transition between different computational states. Such dysfunction may be linked to a range of unhealthy phenotypes, not only involving classical excitation-inhibition (E/I) imbalances or overexcitation—which can lead to epileptic activity or disrupted synchrony—but also affecting broader cognitive and behavioral symptoms.

*Rett Syndrome* is one neurodevelopmental disorder where bipolar VIP+ interneurons could possibly play a significant role in the future. Rett Syndrome, primarily affecting females, is characterized by seizures, intellectual disabilities, and features of autism spectrum disorder (ASD). It is caused by mutations in the MECP2 gene on the X chromosome, which is expressed in all cortical interneurons, including PV+, SST+, and VIP+ types (Mossner et al., 2020). Selective deletion of MECP2 in VIP+ interneurons in male mice models resulted in a few Rett-like symptoms, such as abnormal social behavior, though seizures were not observed (Mossner et al., 2020).

*Dravet Syndrome* is another early-onset neurodevelopmental disorder associated with various symptoms, including seizures, epileptic activity, unexpected death at early development and behavioral issues resembling intellectual disabilities or ASD. Dravet Syndrome is caused by mutations in the SCN1A gene, which encodes the Na + voltage-gated channel subunit Nav1.1. This channel is expressed at the axon initial segment (AIS) of all cortical interneurons, including PV+ and SST+ populations (Goff and Goldberg, 2021). Recent studies in mouse models have indicated that VIP+ interneurons also express Nav1.1 in their AIS. In Dravet Syndrome animal models, VIP+ interneurons exhibited abnormal action potential generation due to reduced Nav1.1 (Goff and Goldberg, 2019). Notably, VIP interneuron-specific SCN1A deletions replicated a number of Dravet syndrome symptoms but did not induce seizures, suggesting that VIP+ interneurons might be involved in managing behavior and network activity (Goff et al., 2023). Conversely, NDNF+ interneurons showed normal Nav1.1 expression and activity in these mouse models, underscoring the distinct roles of different interneuron types in animal models that mimic some symptoms of Dravet Syndrome and highlighting VIP+ interneurons as potential future therapeutic targets.

*Neuropsychiatric Disorders* such as major depression, bipolar disorder, schizophrenia, and addiction also display dysfunctional behavior similar to ASD and intellectual disabilities. In schizophrenia, disrupted prefrontal cortex (PFC) activity has been linked to nicotinic acetylcholine receptor (nAChR) dysfunction. VIP+ cell-mediated inhibition of SST+ cells was impaired in a mouse model carrying a human polymorphism in the α5 nicotinic receptor subunit, associated with nicotine addiction and schizophrenia (Koukouli et al., 2017). Altered nicotinic receptor function in VIP+ cells led to suppressed pyramidal neuron activity and schizophrenia-like hypofrontality. Furthermore, optogenetic activation of VIP+ interneurons improved performance on behavioral tasks and enhanced action plan representations (Kamigaki and Dan, 2017). Thus, modulating VIP+ and/or SST+ interneuron activity holds therapeutic potential for addressing GABAergic hypofunction in schizophrenia.

*Novelty Seeking and Impulsivity* are critical risk factors for schizophrenia and addiction. Recent research showed that ablation of VIP+ interneurons increased impulsive behavior in mice (Hatter and Scott, 2023). Additionally, VIP+ interneuron dysfunction has been linked to F*ragile X Syndrome* (FXS), which is characterized by attention deficits and hypersensitivity to sensory inputs. In FXS mice, VIP+ interneurons exhibited reduced modulatory influence on L2/3 pyramidal neuron activity, leading to impaired performance during distractor tasks (Rahmatullah et al., 2023).

Finally, *Chronic Pain* has also been associated with cortical dysfunction. Recent work found that peripheral nerve injury-induced neuropathic pain reduced VIP interneuron activity in the PFC. This reduction led to decreased pyramidal neuron activity in the PFC, affecting both local network processing and output to the anterior cingulate cortex (ACC). Consequently, decreased PFC PC activity led to reduced glutamatergic transmission in ACC interneurons, increasing ACC pyramidal neuron firing (Li et al., 2022).

Overall, these findings underscore a novel potential role of VIP+ interneurons in behavioral dysfunction and various disorders, suggesting that disruptions in specific inhibitory populations may alter cortical network computations in ways that extend beyond traditional models of excitation-inhibition balance and spiking stability. Future research should investigate the role of NDNF+ interneurons in disease, as emerging evidence suggests their significant impact on cortical processing and behavior. Understanding the specific contributions of NDNF+ interneurons will be critical in uncovering their involvement in neurodevelopmental and neuropsychiatric disorders (Abs et al., 2018; Cohen-Kashi Malina et al., 2021; Hartung et al., 2024; Liebergall and Goldberg, 2024).

## Discussion

In this perspective, we explore the broader implications of interneuronal function and dysfunction in cortical processing. We focus on how distinct types of inhibitory interneurons contribute to network dynamics, their roles in modulating computational states, and the potential consequences of their dysfunction in various neurological and psychiatric disorders.

### Functional roles of inhibitory interneurons

The classical roles of inhibitory interneurons in cortical processing—regulating stability, rhythmicity, and refining cortical representations—are well-documented (Lourenço et al., 2020; Tremblay et al., 2016). These functions are primarily supported by potent inhibitory interneurons such as the PV+ and SST+ cells, which play a crucial role in controlling network activity (Tremblay et al., 2016). However, recent findings suggest that some interneuronal populations, particularly VIP+ and NDNF+ interneurons, may serve non-classical roles. Bipolar VIP+ interneurons, which primarily target other inhibitory interneurons, and NDNF+ interneurons, which modulate pyramidal cell activity through slow GABAA and GABAB-mediated currents, do not fit neatly into the classical framework. Instead, they appear to be crucial in modulating network states in response to behavioral and arousal changes (McGinley et al., 2015a).

### Computational modulation by interneurons

The evidence discussed highlights that bipolar VIP+ and NDNF+ interneurons are integral in modulating the network’s computational properties. Bipolar VIP+ interneurons, through disinhibitory mechanisms, and NDNF+ interneurons, by modulating activity through slower GABAergic mechanisms, significantly impact the network’s ability to shift between different computational states (Hartung et al., 2024; Muñoz et al., 2017). This modulation can alter the network’s encoding of sensory information, shifting from sparse and reliable representations in a “quiet state” to dense and high-gain representations in an “active state” (Figure 2). Such state-dependent processing allows the cortex to adaptively modulate its response to incoming stimuli based on behavioral context, optimizing sensory processing and cognitive functions. The theoretical models discussed demonstrate how these interneuronal circuits enable transitions between different network activity regimes. For instance, the presence of disinhibitory circuits facilitates transitions between afferent-dominated and recurrent-dominated regimes, each characterized by distinct computational properties. This ability to switch between states supports the cortex’s capacity to encode complex patterns or detect weak stimuli, depending on the behavioral demands (Zerlaut et al., 2019).

### Implications for neurological and psychiatric disorders

The implications of interneuronal dysfunction are profound, as evidenced by the association of VIP+ interneurons with various neurological and psychiatric disorders (Goff and Goldberg, 2021). In conditions such as Rett Syndrome and Dravet Syndrome, specific interneuronal populations exhibit dysfunction that impacts behavioral and cognitive outcomes. For instance, VIP+ interneurons in Dravet Syndrome show altered action potential generation and impaired network dynamics, leading to behavioral symptoms but not necessarily seizures. This points to the crucial role of VIP+ interneurons in maintaining network stability and behavioral regulation, highlighting their potential as therapeutic targets. Similarly, disorders like schizophrenia and Fragile X Syndrome illustrate the impact of interneuronal dysfunction on cognitive and behavioral phenotypes (Rahmatullah et al., 2023). Schizophrenia models reveal that altered VIP interneuron function affects pyramidal neuron activity and prefrontal cortex processing, potentially leading to symptoms such as hypofrontality (Koukouli et al., 2017). In Fragile X Syndrome, VIP interneurons show reduced modulatory influence, affecting sensory processing and task performance (Rahmatullah et al., 2023). These findings underscore the role of specific interneuron types in modulating cortical computations and highlight the need for targeted interventions to address their dysfunction. The connection between cortical dysfunction and chronic pain further emphasizes the broader implications of interneuronal activity. VIP interneurons’ role in pain processing and their impact on the anterior cingulate cortex illustrate how changes in cortical interneuron function can affect both local network dynamics and broader sensory processing pathways.

### Future directions

#### Interneuronal modulation of cortical computations through plasticity regulation

Cortical interneurons play a pivotal role in modulating synaptic plasticity, a fundamental mechanism for learning and memory. Emerging evidence highlights that specific interneuronal subtypes can either promote or suppress plasticity depending on their connectivity and functional properties (Agnes and Vogels, 2024; Capogna et al., 2021; Lamsa et al., 2007; Tzilivaki et al., 2023). The modulation of cortical computations by interneurons can be further examined through the lens of plasticity regulation. Interestingly, the interneuronal populations highlighted in this perspective predominantly influence the membrane potential dynamics in the apical tufts of pyramidal cells. This occurs indirectly via SST+ projections and directly through GABAergic inhibition mediated by bipolar VIP+ and NDNF+ interneurons. These apical dendritic compartments are critical sites for calcium-dependent depolarized plateau potentials, which are strongly implicated in synaptic plasticity processes (Magee and Grienberger, 2020). Beyond their role in pyramidal neuron plasticity, recent findings reveal that interneurons themselves, such as PV+ and SST+ populations, are capable of active dendritic processing due to their nonlinear dendrites (Chiovini et al., 2014; Cornford et al., 2019; Katona et al., 2011; Morabito et al., 2024; Tzilivaki et al., 2019, 2022). This plasticity in interneurons is another layer of complexity that may profoundly influence cortical computations. It would be particularly intriguing to investigate how modulation by bipolar VIP+ and NDNF+ interneurons in the cortex could regulate the plasticity of other interneurons, such as PV+ and SST+. Such interactions could potentially reshape the nonlinear dendritic computations not only of pyramidal neurons but also of these interneurons, further enhancing or diversifying the cortical network’s computational repertoire. This spatial and functional convergence suggests a possible link between learning mechanisms and behavioral states, with inhibition serving as a shared regulatory gate. Such insights raise a fundamental question: does learning occur preferentially during specific behavioral states? Future research should aim to dissect the functional role of these interneuronal circuits and the inhibition-dependent mechanisms underlying this intriguing relationship. Understanding this connection could illuminate how the brain seamlessly integrates plasticity with behavioral adaptability and offers an exciting avenue for uncovering how dendritic processing in both pyramidal neurons and interneurons contributes to dynamic cortical computations.

#### Novel experimental paradigms to study inhibitory modulations of computations

This perspective underscores the need for innovative experimental paradigms that can capture both the computational modulations within cortical networks and their dependence on inhibitory mechanisms. In sensory systems, the traditional approach often involves designing cortex-dependent behavioral tasks to study performance as a function of behavioral states (McGinley et al., 2015a, 2015b). However, this approach falls short of fully characterizing the ability of cortical networks to alter their computational properties across behavioral states. Typically, such studies only demonstrate that one behavioral state optimizes performance for a single task, without addressing the broader adaptability of network processing. To establish that cortical state transitions lead to dynamic changes in computational properties, future studies must employ multi-task paradigms. These protocols should involve at least two tasks, each optimized for different behavioral states, and demonstrate that performance fluctuations align with the state-dependent properties of the cortex. For instance, in visual processing, sparse and high-reliability firing modes during intermediate arousal states (Figure 2) might favor the encoding of natural scenes, while dense, high-gain firing modes during heightened arousal could optimize the detection of faint visual cues. Recent experimental findings already hint at such differential encoding properties across behavioral states in the mouse visual cortex (Neske et al., 2019). Designing such multi-task paradigms presents significant challenges, particularly in identifying task pairs that distinctly capitalize on the computational benefits of different cortical states. Here, theoretical models can play a pivotal role. By simulating distinct network computational properties, these models can guide the design of stimuli and tasks tailored to highlight state-dependent processing advantages. Furthermore, these paradigms must be paired with interventional tools such as optogenetics or chemogenetics to directly test the involvement of specific interneuronal populations in modulating state-dependent processing of sensory signals. The integration of experimental and computational approaches is not merely complementary but essential. Computational models can bridge the gap between theory and experiment, refining hypotheses and helping to interpret complex data, while experimental validation ensures biological relevance. Together, these approaches offer a powerful framework for elucidating the nuanced role of inhibitory modulations in cortical computations and advancing our understanding of the dynamic interplay between behavior, neural states, and network function.

## Conclusion

The findings discussed underscore the intricate roles of interneurons in cortical processing. While the classical functions of inhibition—such as maintaining network stability and refining sensory representations—are well-documented, emerging evidence reveals that specific interneuronal subpopulations contribute to dynamic computational modulation and processing in ways that surpass traditional models. Future research must focus on deciphering the precise mechanisms through which various interneuronal types impact network states and behavior. A crucial first step in this endeavor is to comprehensively characterize the cellular properties of these interneuronal populations. Recent studies, particularly from the hippocampus, suggest that PV+ and NDNF+ interneurons exhibit a range of plasticity mechanisms and support active nonlinear integration within their complex dendritic structures (Kullmann and Lamsa, 2007; Mercier et al., 2019; Tzilivaki et al., 2022, 2023). Moreover, emerging data from the somatosensory cortex indicate that PV+ and SST+ interneurons demonstrate differential nonlinear behaviors that contribute to their specific roles in temporal control of cortical dynamics (Morabito et al., 2024). Plasticity induction has also been recently studied in L1 cortical NDNF+ interneurons (Abs et al., 2018). However, it remains to be determined whether similar plasticity and dendritic properties are present in other interneuronal types, such as VIP+ interneurons. Understanding the molecular and circuit-level alterations associated with interneuronal dysfunction is essential for developing targeted therapies for related disorders. Additionally, it is vital to explore how these insights can be applied to human pathology and behavior. Such research will be crucial for advancing our comprehension of cortical function and its implications for neurological and psychiatric conditions.

## Data Availability

The original contributions presented in the study are included in the article/supplementary material, further inquiries can be directed to the corresponding authors.
